# Role of Leukotrienes and Leukotriene Modifiers in Asthma

**DOI:** 10.3390/ph3061792

**Published:** 2010-06-02

**Authors:** Paolo Montuschi

**Affiliations:** Department of Pharmacology, Faculty of Medicine, Catholic University of the Sacred Heart, Largo Francesco Vito, 1-00168 Rome, Italy; E-Mail: pmontuschi@rm.unicatt.it; Tel: +39-06-30156092; Fax: +39-06-30156292

**Keywords:** asthma, leukotrienes, cysteinyl-leukotrienes, leukotriene B_4_, leukotrienes receptor antagonists, inhaled glucocorticoids, airway inflammation

## Abstract

Leukotrienes (LTs), including cysteinyl LTs (CysLTs) and LTB_4_, are potent lipid mediators that are pivotal in the pathophysiology of asthma phenotypes. At least two receptor subtypes for CysLTs – CysLT_1_ and CysLT_2_ – have been identified. Most of the pathophysiological effects of CysLTs in asthma, including increased airway smooth muscle activity, microvascular permeability and airway mucus secretion, are mediated by the activation of the CysLT_1_ receptor. LTB_4_ may have a role in the development of airway hyperresponsiveness, severe asthma and asthma exacerbations. Although generally less effective than inhaled glucocorticoids, CysLT_1_ receptor antagonists can be given orally as monotherapy in patients with persistent mild asthma. In patients with more severe asthma, CysLT_1_ receptor antagonists can be combined with inhaled glucocorticoids. This therapeutic strategy improves asthma control and enables the dose of inhaled glucocorticoids to be reduced, while maintaining similar efficacy. The identification of subgroups of patients with asthma who respond to CysLT_1_ receptor antagonists is relevant for asthma management, as the response to these drugs is variable. The potential anti-remodeling effect of CysLT_1_ receptor antagonists might be important for preventing or reversing airway structural changes in patients with asthma. This review discusses the role of LTs in asthma and the therapeutic implications of the pharmacological modulation of the LT pathway for asthma.

## 1. Introduction

Leukotrienes (LTs), including cysteinyl-LTs (LTC_4_, LTD_4_, and LTE_4_) and LTB_4_, are potent biological lipid mediators derived from arachidonic acid through the 5-lipoxygenase (5-LO) pathway [1,2,3,4,5]. Specific pathways for the synthesis of cysteinyl-LTs from arachidonic acid are present in several types of inflammatory cells and become activated during allergic airway inflammation [3,5]; moreover, other cell types like platelets and endothelial cells have a unique capacity to produce large amounts of cysteinyl-LTs from the chemically reactive intermediate LTA_4_ via intercellular transfer mechanisms [5]. 

Leukotrienes play a central pathophysiological role in asthma [1,2,3,4,6], particularly in specific subgroups of patients with asthma. Cysteinyl-LTs induce pathophysiological responses similar to those associated with asthma and elevated cysteinyl-LT concentrations have been detected in biological fluids, including bronchoalveolar lavage (BAL) [7], sputum [8], and exhaled breath condensate (EBC) from patients with asthma [9,10]. The cysteinyl-LTs are likely to contribute to airway remodelling that characterises persistent asthma [11,12]. 

Two G-protein coupled receptor subtypes for cysteinyl-LTs (CysLT_1_ and CysLT_2_) have been identified [13,14]. Most of the effects of cysteinyl-LTs relevant to the pathophysiology of asthma are mediated by activation of the CysLT_1_ receptor [2,3], which is expressed in different types of inflammatory and structural cells in the airways [13,15]. 

The most convincing evidence for an etiological role of cysteinyl-LTs in asthma comes from the therapeutic efficacy of CysLT_1_ receptor antagonists (e.g., montelukast, zafirlukast, pranlukast), commonly known as leukotriene receptor antagonists (LTRAs), and 5-lipoxygenase (5-LO) inhibitors (e.g., zileuton) in patients with asthma [4]. These drugs are effective in preventing asthmatic responses induced by allergen-challenge [16], exercise [17], and aspirin [18]. Moreover, CysLT_1_ receptor antagonists have a therapeutic role in persistent asthma as they improve pulmonary function, symptoms and quality of life, and reduce β-agonist use, airway and peripheral eosinophilia, asthma exacerbations, and the required dose of inhaled corticosteroids in asthma patients [19,20].

CysLT_1_ receptor antagonism has anti-remodeling effects in the airways in an animal model of human asthma [21] and inhibitory effects on airway structural cells that are functionally involved in airway remodeling in allergic airway inflammation in patients with asthma [22]. 

As a potent chemoattractant for neutrophils, LTB_4_ can have a central role in the neutrophilic inflammation that characterises severe asthma and asthma exacerbations [23], whereas its role in mild to moderate persistent asthma is less known. Elevated LTB_4_ concentrations in EBC have been reported in adults and children with stable asthma [24,25,26,27,28]. The lack of effect of LTB_4_ receptor antagonists in allergen-induced early or late phase airway obstruction in patients with asthma [29] argues against an important role for LTB_4_ in acute bronchoconstriction in asthma. However, a role for LTB_4_ in airway hyperresponsiveness (AHR) in asthma has been proposed [30,31,32].

This review will examine the role of leukotrienes in asthma and the therapeutic implications of the leukotriene pathway inhibition for asthma.

## 2. Biosynthesis and Metabolism of Leukotrienes

**Scheme 1:**
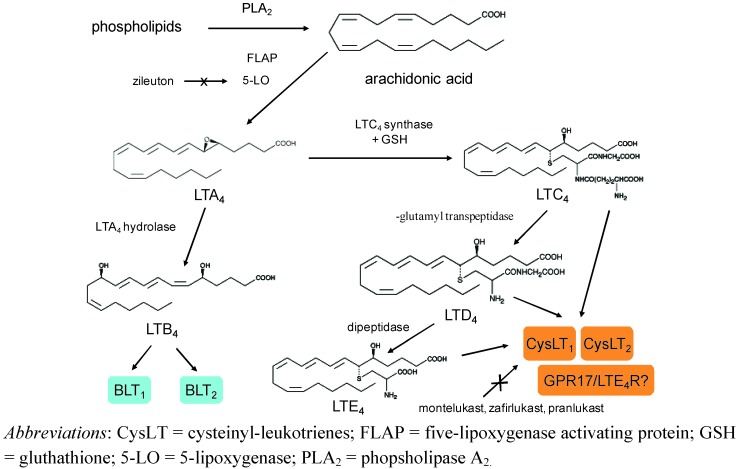
Biosynthetic pathway of leukotrienes (LTs), LT receptors, and mechanisms of action of antileukotriene drugs (reproduced with permission from reference [2]).

Leukotrienes derive from 5-LO activity (Scheme 1). Arachidonic acid, that is esterified on plasma membrane phospholipids, is cleaved by the action of different phopsholipase A_2_ enzymes, released and metabolized into LTA_4_. This leukotriene is subsequently metabolized by LTA_4_ hydrolase into LTB_4_ and, into LTC_4_ by LTC_4_ synthase or different members of the membrane-associated proteins in the eicosanoid and glutathione metabolism superfamily (MAPEG), including microsomal glutathione transferase 2 (MGST2) [5]. LTC_4_ in turn is metabolized by a γ-glutamyl transpeptidase into LTD_4_ that is then metabolized by a dipeptidase into LTE_4_. LTA_4_ is highly reactive, with an estimated half-life < 3 seconds [5]. LTC_4_ and its metabolites, LTD_4_ and LTE_4_, are known as cysteinyl-LTs due to the common cysteine in their side chains. Biosynthesis of LTs requires cellular activation, including IgE receptor cross-binding on mast cell surface, and involves a five-lipoxygenase activating protein (FLAP) that binds 5-LO and facilitates the metabolism of arachidonic acid [2,3,5]. The intracellular distribution of 5-LO varies between different cells. 5-LO is mainly expressed in granulocytes, monocytes, macrophages, mast-cells and B lymphocytes [3]. Mast cells and eosinophils can produce large amounts of LTC_4_ from an endogenous pool of arachidonic acid. Human bronchial fibroblasts constitutively express 5-LO, FLAP, LTA_4_ hydrolase, and LTC_4_ synthase and produce cysteinyl-LTs and LTB_4_ spontaneously *in vitro* [33]. Cells that do not express 5-LO, including platelets, erythrocytes, endothelial cells and epithelial cells, also have the capacity to produce cysteinyl-LTs and/or LTB_4_ through the transcellular metabolism of LTA_4_ synthesized by activated neutrophils [5]. After their intracellular formation, cysteinyl-LTs and LTB_4_ are released to the extracellular space through specific carrier-proteins that are potential targets for future antileukotriene drugs [3]. 

## 3. Receptors and Mechanism of Action of Leukotrienes

Two G-protein coupled receptor subtypes for cysteinyl-LTs (CysLT_1_ and CysLT_2_), that have 38% amino acid identity, have been identified [13,14] (Scheme 1). There is evidence that supports the existence of distinct CysLT receptors [34,35,36,37,38]. Increased vascular permeability induced by LTE_4_ in mice lacking CysLT_1_ and CysLT_2_ receptors suggests the existence of a third cysLT receptor that responds preferentially to LTE_4_ [34]. A G-protein-coupled receptor (GPCR) GPR17, that responds both to cysteinyl-LTs and to uracil nucleotides [38], is a ligand independent, constitutive negative regulator for the CysLT_1_ receptor and suppresses CysLT_1_ receptor-mediated function at the cell membrane [35]. Most of the effects of cysteinyl-LTs relevant to the pathophysiology of asthma are mediated by activation of the CysLT_1_ receptor [1,2] that is expressed in monocytes and macrophages, eosinophils, basophils, mast cells, neutrophils, T cells, B lymphocytes, pluripotent hemopoietic stem cells (CD 34^+^), airway smooth muscle cells, bronchial fibroblasts, and vascular endothelial cells [13,15,33]. The CysLT_2_ receptor is expressed in human peripheral basophils [39], endothelial cells [40], cultured mast cells [14], and in nasal eosinophils and mast cells in patients with active seasonal allergic rhinitis [41]. In human cultured mast cells, CysLT_2_ activation may elicit IL-8 generation with potential neutrophilic inflammation [14] that is a characteristic of acute and severe asthma. Expression of CysLT_2_ receptors on eosinophils is increased in patients with asthma exacerbations, especially in nonatopic subjects, and is up-regulated by interferon-γ indicating a role for this receptor subtype in acute asthma [42]. At present, the role of the CysLT_2_ receptor in allergic inflammation is largely unknown [40]. CysLT_1_ and CysLT_2_ receptor activation involves increased intracellular calcium [13,43], but the complete signal transduction pathway is not known. In cell lines derived from humans and monkeys, protein kinase C activity is the principal regulator of both rapid agonist-dependent internalization and rapid agonist-dependent desensitization [43].

Two LTB_4_ receptor subtypes (BLT_1_ and BLT_2_), that are cell surface G protein-coupled seven transmembrane domain receptors, have been identified [44,45]. Both receptor subtypes are expressed in a human mast cell line (HMC-1) [46]. BLT_1_ receptors are expressed in human bronchial fibroblasts [33] and in a subset of effector memory IL-13-producing CD8^+^ T cells in bronchoalveolar lavage fluid of patients with asthma [47]. BLT_1_ expression on Ag-primed T cells [48] and dendritic cells [31] is required for the development of AHR in mice, indicating a possible role for LTB_4_ in AHR in patients with asthma. 

## 4. Biological Effects of Leukotrienes in the Airways

Cysteinyl-LTs induce pathophysiological responses that are observed in patients with asthma [1,2,3]. Cysteinyl-LTs are the most potent endogenous bronchoconstrictors. LTC_4_, LTD_4_, and LTE_4_ have similar contractile activity on human airway smooth muscle *in vitro*. This effect has been confirmed by bronchoprovocation studies in healthy subjects [3]. Patients with asthma are hyperresponsive to inhalation of LTC_4_, LTD_4_, and LTE_4_ [3]. Cysteinyl-LTs increase mucus secretion in isolated animal and human airways and increase microvascular permeability in the lungs in experimental animals [3]. These effects can contribute to bronchial obstruction in patients with asthma. Cysteinyl-LT inhalation in patients with asthma increases the number of sputum eosinophils and causes recruitment of eosinophils into the airway mucosa [49]. However, the mechanism(s) of the eosinophil chemotactic effect induced by cysteinyl-LTs in not completely known. 

In addition to their local effects in the airways, cysteinyl-LTs have several effects that contribute to the inflammatory processes characterising asthma [4,50]. Cysteinyl-LTs (1) modulate leucopoiesis induced by granulocyte-macrophage colony stimulating factor, interleukin (IL)-5, and IL-3 and prime progenitor cells to differentiate into mature blood cells; (2) induce leukocyte migration from the bone marrow into the circulatory system; (3) cause chemotaxis of eosinophils increasing their cellular adhesion and transendothelial migration accross the vessel wall into the airways; (4) increase eosinophil survival in response to mast cell and lymphocyte paracrine signals; (5) activate eosinophils, mast cells, T lymphocytes, monocytes and basophils [4,50]. Cysteinyl-LTs have a central role in lung inflammation induced by allergen challenge as shown by the reduced Th_2_ cell-dependent inflammatory response in LTC_4_ synthase null mice [51].

Cysteinyl-LTs are functionally involved in airway remodeling that includes eosinophil cell inflammatory response, airway smooth muscle cell hyperplasia, mucus gland hyperplasia, mucus hypersecretion, and collagen deposition beneath the epithelial layer and in the lung interstitium at sites of leukocytes infiltration [11,12]. Montelukast reduces allergen-induced lung inflammation and fibrosis in an animal model of the airway remodeling changes observed in patients with persistent asthma [21].

LTB_4_ may contribute to a reduction in airway calibre due to local edema and increasing mucus secretion, although it has no bronchoconstrictor effect in healthy and asthmatic subjects [2,3]. As it is a potent chemoattractant for neutrophils, LTB_4_ might be functionally involved in the neutrophilic phenotype of asthma that characterizes patients with severe asthma [23] or asthma exacerbations. Persistently elevated LTB_4_ concentrations in plasma in children with asthma exacerbation at least one month after the acute episode [52], elevated LTB_4_ concentrations in EBC in adults with mild asthma [24], and elevated LTB_4_ concentrations in EBC in children with mild-to-moderate persistent asthma [26] could indicate a pathophysiological role of LTB_4_ in chronic stable asthma of lesser severity [53]. However, the pathophysiological role of LTB_4_ in mild-to-moderate persistent asthma in not completely known and requires further studies. In mice, LTB_4_ has an essential role in triggering airway allergic responses by activating BLT_1_ receptors on a subset of effector CD8^+^ T cells [47]. The absence of BLT_1_ receptors or their antagonism on these cells markedly reduces allergen challenge-induced AHR and airway inflammation in mice [31,47,48]. A subset of CD8^+^ T cells expressing BLT_1_ receptors have been identified in BAL and lung tissue from subjects with asthma, but not from healthy subjects [47]. The number of this subset of CD8^+^ T cells is increased in patients with steroid-resistant asthma compared with those with steroid-sensitive asthma [47], indicating a possible role for BLT_1_ receptors in steroid response. However, the biological significance of LTB_4_-induced activation of effector CD8^+^ T cells in patients with asthma needs to be established. A role for LTB_4_ in AHR is also suggested by the fact that chronic treatment with zileuton, that reduces synthesis of both cysteinyl-LT and LTB_4_, decreases AHR in asthmatic patients [54,55], concomitant with a reduction of *ex vivo* LTB_4_ production [55]. In contrast, selective CysLT_1_ antagonists have only a modest effect on AHR [4,56]. 5-LO inhibition is very effective in causing chronic improvement in nasal function in patients with aspirin-sensitive asthma (ASA) at baseline [54], whereas CysLT_1_ receptor antagonists, that significantly reduce bronchospastic response, have only minor effects on ASA-induced upper airway reactions [57]. These data indicate that LTB_4_ can have a pathophysiological role in nasal symptoms in ASA. Alternatively, or in addition to that, nasal symptoms in patients with ASA could be due to activation of CysLT_2_ receptors or distinct LTE_4_ receptors [34,35,36,37,38]. Although LTE_4_ has little activity at CysLT_1_ and CysLT_2_ receptors [37], inhalation of LTE_4_ increases airway inflammatory cells [58,59] and AHR in asthma patients [60], particularly in those with ASA [37]. In sensitized mice, intranasal LTE_4_ potentiates pulmonary inflammation in response to low-dose aerosolized antigen [36]. This effect persists in mice lacking both CysLT_1_ and CysLT_2_ receptors but not in mice lacking P2Y_12_ receptors, indicating that the P2Y_12_ receptor is required for pro-inflammatory effects of LTE_4_ [36].

## 5. Measurement of LTs in Biological Fluids in Patients with Asthma

LTs have been measured in exhaled breath condensate (EBC) [9,10,24,25,26,27,28,61,62,63,64], sputum [27,65], BAL fluid [7], and urine [66,67,68] from asthmatic patients. There are several reports of increased LT levels in EBC in both adults and children with asthma [9,10,24,25,26,27,28,61,62,63,64,69], but the methodology used requires standardization [70]. Sputum CysLT concentrations are elevated in patients with asthma, reflecting asthma severity [65]. LT concentrations are increased in BAL fluid in patients with asthma, including those with nocturnal asthma [7]. Measurement of LTs in BAL fluid, sputum and EBC is likely to reflect pulmonary synthesis of LTs. 

Urinary measurement of LTE_4_, the most abundant CysLT excreted in the urine, is used for assessing the systemic synthesis of CysLTs as circulating concentrations of LTs are usually undetectable [66]. No or only slight differences in urinary LTE_4_ concentrations between healthy and atopic asthmatic subjects have generally been reported under basal conditions [66]. In contrast, urinary LTE_4_ excretion is elevated after allergen challenge in atopic asthmatics [3,66], in aspirin-sensitive asthmatics under basal conditions [67], in patients with nocturnal asthma [7], in severe asthma [71], and during asthma exacerbations [68].

## 6. Effects of Leukotriene Receptor Antagonists in Asthma

Selective CysLT_1_ receptor antagonists that have been approved for clinical use in asthma include montelukast, zafirlukast and pranlukast (Table 1). Zileuton, a 5-LO inhibitor, has been approved for the prevention and chronic treatment of asthma in adults and children 12 years of age and older in the United Kingdom and USA (Table 1). Montelukast is the most prescribed CysLT_1_ receptor antagonist in Europe and the USA, whereas pranlukast is only marketed in Japan and other Asian countries. Zafirlukast was the first anti-LT that was approved in Europe, but it is not frequently prescribed due to possible food and drug interactions, and its twice daily administration regimen [2,3]. The fact that selective CysLT_1_ receptor antagonists and 5-LO inhibitors have similar efficacy in short-term treatment studies and challenge models indicates that most of the antiasthmatic effects of anti-LTs are due to CysLT_1_ antagonism [3]. The use of zileuton is limited because of a small, but distinct, incidence of hepatic enzyme elevation, which is not observed with montelukast, and the short half-life, requiring four daily administrations [3]. A twice-daily controlled-release formulation of zileuton has been approved by the U.S. Food and Drug Administration (FDA) [1].

At least two aspects of selective 5-LO inhibitors concerning the inhibition of LTB_4_ synthesis deserve further investigation: their effects on AHR in patients with asthma [54,55], that is slightly affected by CysLT_1_ antagonists [4]; the potential efficacy of 5-LO inhibitors in rhinitis and rhinopolyposis as these drugs are very effective in reducing nasal symptoms in patients with ASA [54].

**Table 1:** Main pharmacological characteristics of antileukotrienes (reproduced with permission from reference [2]).

Drug	Mechanism of action	Indication	Benefits	Side effects	Dose	Comments
Montelukast	CysLT_1_ receptor antagonism	asthma, allergic rhinitis	as monotherapy in children with mild persistent asthma; particularly effective in exercise-induced asthma, ASA, allergen-induced asthma; as add-on therapy with ICS	headache, abdominal pain; possible association with Churg-Strauss syndrome	adults: 10 mg o.d. children 6 to 14 years of age: 5 mg o.d. children 2 to 5 years of age: 4 mg o.d.	most widely prescribed CysLT_1_ receptor antagonist
Pranlukast	CysLT_1_ receptor antagonism	asthma, allergic rhinitis	particularly effective in exercise-induced asthma, ASA, allergen-induced asthma; as add-on therapy with ICS	abdominal pain, liver enzymes elevations; possible association with Churg-Strauss syndrome	adults: 225 mg b.i.d.	only marketed in Asia
Zafirlukast	CysLT_1_ receptor antagonism	asthma	particularly effective in exercise-induced asthma, ASA, allergen-induced asthma; as add-on therapy with ICS	headache, abdominal pain, liver enzymes elevations; possible association with Churg-Strauss syndrome	children ≥ 12 years of age and adults: 20 mg b.i.d. children 5 to 11 years of age: 10 mg b.i.d.	first CysLT_1_ receptor antagonist to be approved; food and drug interactions
Zileuton	5-LO inhibition	asthma	particularly effective in exercise-induced asthma and ASA	headache, abdominal pain; liver enzymes elevations (5%)	adults and children 12 years of age and older: 600 mg q.i.d.	virtually abandoned because of poor compliance and hepatic toxicity

*Abbreviations:* ASA = aspirin-sensitive asthma; CysLT = cysteinyl-leukotrienes; ICS = inhaled corticosteroids.

CysLT_1_ receptor antagonists improve symptoms and lung function, and reduce exacerbation rate, the use of rescue β_2_ bronchodilators, and airway and blood eosinophilia in adults and children with asthma of different severity [1,2,3,4]. Cys-LT_1_ receptor antagonists provide a prompt improvement in asthma control, although low-dose inhaled glucocorticoids are generally more effective than Cys-LT_1_ receptor antagonists as first-line maintenance therapy for patients with persistent asthma who are undertreated and remain symptomatic while taking short-acting β_2_-agonists alone [20]. When added to standard therapy in adults with asthma exacerbations, intravenous montelukast (7 mg) significantly improves airway obstruction throughout the 2 hours immediately after administration, with an onset of action as early as 10 minutes, indicating a possible therapeutic role for CysLT_1_ receptor antagonists in severe acute asthma [72,73]. CysLT_1_ receptor antagonists are effective in reducing early and late asthmatic responses induced by allergen inhalation [16,74]. Unlike budesonide, montelukast inhibits the maximal early asthmatic response, whereas both drugs attenuate the late asthmatic response [16]. However, anti-LTs reduce allergen-induced AHR to a lesser extent than do inhaled glucocorticoids [16]. This could be explained by the fact that AHR is multifactorial and relatively independent of the acute inflammatory response mediated by LTs. Moreover, inhaled glucocorticoids inhibit several airway inflammatory cells and mediators that are pivotal in the AHR pathophysiology, whereas anti-LTs selectively block LT-mediated eosinophilic inflammation [16]. CysLT_1_ receptor antagonists are also effective in reducing allergen-induced asthmatic response in children [75]. Montelukast given once daily at a dose of 10 mg protected against exercise-induced broncho-constriction over a 12-week period in adults with asthma [17]. Treatment with CysLT_1_ receptor antagonists reduces the time to recovery from the maximal decrease in FEV_1_, the maximal decrease in FEV_1_, and the area under the FEV_1_ *versus* time curve after exercise [17]. These effects are observed as soon as two hours after a single oral dose of montelukast (10 mg) and are maintained up to 24 hours [76,77]. Montelukast was superior to salmeterol in the chronic treatment of exercise-induced bronchoconstriction over a period of eight weeks in adults with mild asthma, as demonstrated by effect size, peristence of effect and higher tolerability during the study period [78]. Likewise, CysLT_1_ receptor antagonists are effective in exercise-induced bronchoconstriction in children [79]. CysLT_1_ antagonism and 5-LO inhibition protect against the reduction in FEV_1_ in response to aspirin challenge [3] and improve asthma control in aspirin-sensitive patients over and above the therapeutic response to glucocorticoids, an effect that is independent of baseline urinary LTE_4_ [18,54].

Some aspects of the clinical pharmacology of CysLT_1_ receptor antagonists deserve further discussion: (1) their role as monotherapy in patients with asthma; (2) their efficacy and the possibility of reducing the dose of inhaled glucocorticoids when addedd to these drugs; (3) the variability in their therapeutic response; (4) their potential anti-remodeling effect in the airways; (5) their safety.

In the USA, monotherapy with CysLT_1_ receptor antagonists is a common therapeutic option for patients with mild asthma [80], although inhaled glucocorticoids are generally preferred [81] as in Europe. However, CysLT_1_ receptor antagonists are less effective than inhaled glucocorticoids as first-line agents in both adults [20] and children with asthma [82].

**Figure 1:**
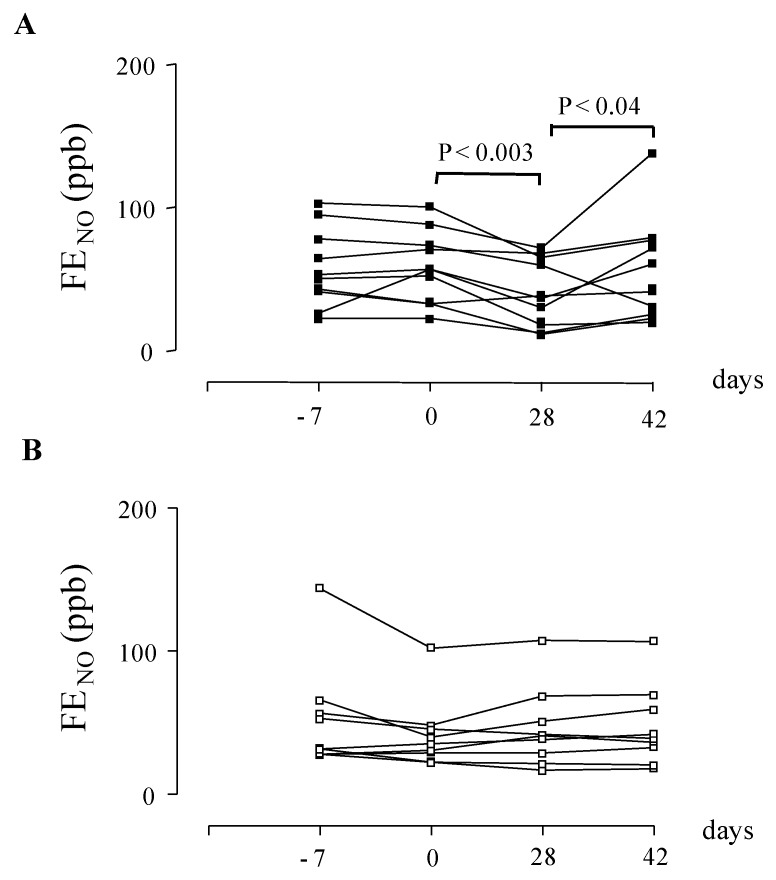
(A) Fractional exhaled nitric oxide (F_E_NO) in children with asthma (n = 14) at baseline (day -7), before treatment with montelukast (filled squares) (day 0), after treatment with oral montelukast (5 mg qd for four weeks) (day 28), and two weeks after montelukast withdrawal (day 42). (B) F_E_NO in children with asthma (n = 12) at baseline (day -7), before treatment with placebo (open squares) (day 0), after treatment with matching placebo (5 mg qd for four weeks) (day 28), and two weeks after placebo withdrawal (day 42). Values are expressed as mean ± SD.

**Figure 2:**
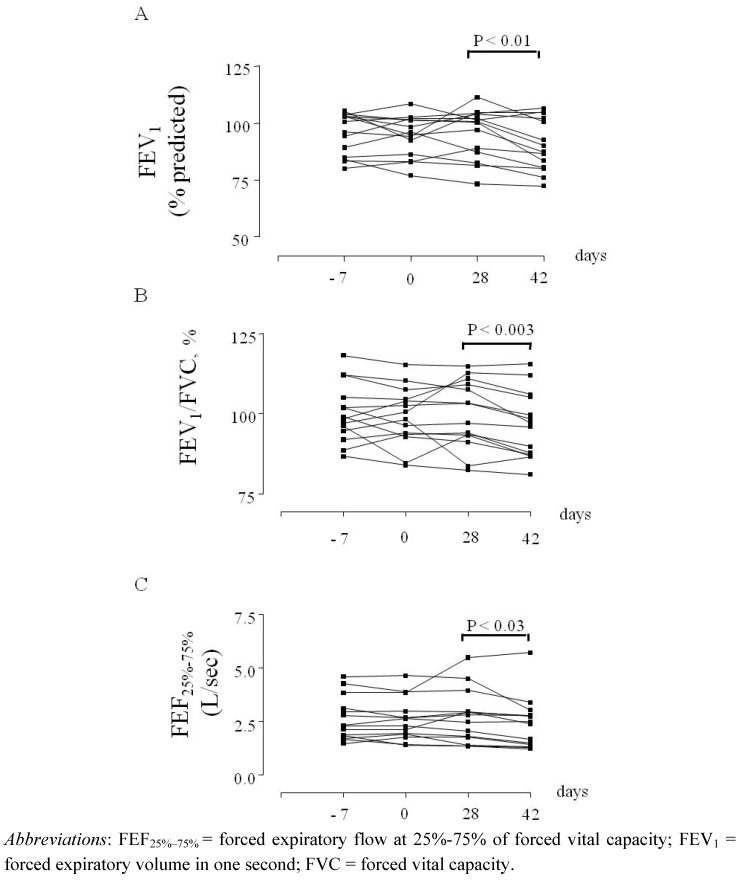
FEV_1_ percentage of predicted values (A), FEV_1_/FVC ratio values (B), and FEF_25%–75%_ values (C) in children with asthma (n = 14) at baseline (day -7), before treatment with montelukast (day 0), after treatment with oral montelukast (5 mg qd for four weeks) (day 28), and two weeks after montelukast withdrawal (day 42).

In Europe, CysLT_1_ receptor antagonists are currently indicated for preventing exercise-induced bronchoconstriction [3]. In patients with asthma who are not sufficiently controlled with a constant dose of inhaled budesonide alone, add-on therapy with montelukast improves asthma control [83] to a level comparable to that achieved by doubling the dose of budesonide [19]. The advantage of this therapeutic strategy would be the reduced risk of side effects due to long-term administration of high-dose inhaled glucocorticoids [19]. In patients whose symptoms remain uncontrolled with inhaled fluticasone alone, the addition of montelukast is a therapeutic option [84], although the addition of a long-acting β_2_-agonist (LABA) is generally more effective than a CysLT_1_ receptor antagonist for preventing exacerbations requiring systemic steroids, and for improving lung function, symptoms and the use of rescue β_2_ agonists [85,86]. In patients with well-controlled asthma based on symptoms and lung function testing, the addition of pranlukast to the combination of inhaled glucocorticoids and LABAs gives better control of airway inflammation compared with therapy with the combination of inhaled glucocorticoid/LABA alone [87,88]. In children with mild persistent asthma, montelukast withdrawal can result in enhanced airway inflammation, as reflected by increased fractional exhaled nitric oxide concentrations (F_E_NO) (Figure 1) and worsening of lung funtion (Figure 2) [89]. Add-on therapy with CysLT_1_ receptor antagonists enables a reduction in the dose of inhaled glucocorticoids required to control asthma [19,90]. As the LT pathway is relatively steroid-resistant [91], the combination of LTRAs and inhaled glucocorticoids can increase therapeutic efficacy in subgroups of patients with asthma who respond to LTRAs.

AHR to LTD_4_, and urinary LTE_4_ concentrations in adults with mild asthma are not affected by inhaled fluticasone (500 μg b.i.d. for two weeks) [91]. Treatment with inhaled fluticasone (100 μg b.i.d. for four weeks) reduces LTE_4_ concentrations in EBC by 18% in children with intermittent and mild persistent asthma [10]. Taken together, this evidence indicates that inhaled glucocorticoids have limited, if any, effects on the biosynthesis of Cys-LTs and AHR to Cys-LTs [91]. 

The therapeutic response to CysLT_1_ receptor antagonists as well as to inhaled glucocorticoids in both adults [19,92,93] and children with asthma is variable [82,92]. Identification of patients who are most likely to respond to LTRAs and/or inhaled glucocorticoids might have important clinical implications, in view of the fact that a tailored, individualized approach to asthma management and assessment is preferable for asthma control than a strategy directed to the best outcome in a group of patients [82]. Some phenotypic features, including higher F_E_NO concentrations, serum IgE and eosinophil cationic protein concentrations, total blood eosinophil counts, lower levels of methacholine provocative concentration (PC)_20_ causing a 20% fall in FEV_1_ and lower levels of pulmonary function, are associated with a therapeutic response to fluticasone in children with asthma [82,92]; a therapeutic response to montelukast is associated with younger age, shorter disease duration, higher urinary LTE_4_ concentrations [82,92] and elevated LTE_4_ concentrations in EBC [9]. Studies on biomolecule profiles in biological fluids and genetic polymorphisms of 5-LO pathway and CysLT receptors [94] could help to predict the therapeutic response to CysLT_1_ receptor antagonists.

In an animal model of asthma, CysLT_1_ receptor antagonists not only prevent allergen-induced airway changes, but also reverse structural changes including subepithelial fibrosis and airway smooth muscle cell layer thickening that are not affected by glucocorticoid treatment [21]. These findings could clarify the role of Cys-LTs in airway remodeling [12] and have important implications for the management of patients with asthma as they might indicate new therapeutic effects of CysLT_1_ receptor antagonists. Inhaled glucocorticoids also reduce basal membrane thickening [95] and subepithelial collagen deposition [96], although these effects seem to have limited impact on the clinical evolution of asthma [97]. In one study, montelukast at a dose of 10 mg once daily for eight weeks reduced myofibroblast accumulation in the airways observed in biopsies of patients with asthma following low-dose allergen challenge [22]. However, whether CysLT_1_ receptor antagonists prevent airway remodeling and/or reverse established airway structural changes in patients with asthma require further research.

CysLT_1_ receptor antagonists are generally considered to be safe and well tolerated, with headache and gastric discomfort being the most common side effects [3]. However, an association between treatment with CysLT_1_ receptor antagonists and severe adverse events including Churg–Strauss syndrome [98] and suicidality [99] has been reported. An etiologic role for CysLT_1_ receptor antagonists in the Churg–Strauss syndrome is generally excluded [3]. However, a recent analysis of the FDA adverse event reporting system database has shown that LTRA therapy was a suspect medication in most confirmed cases of Churg–Strauss syndrome reported [98]. In the majority of cases treated with a LTRA, Churg–Strauss syndrome could not be explained by either glucocorticoid withdrawal or pre-existing Churg–Strauss syndrome [98]. Based on a limited number of postmarketing suicide-related adverse experience reports, the FDA issued a warning raising concerns about the suicidality potential of montelukast and other CysLT_1_ receptor antagonists, and similar changes were submitted to regulatory agencies around the world in October 2007 [99]. A review of the available clinical trial database on montelukast regarding suicidality showed that no completed suicides were reported in any studies [99]; adverse experiences possibly related to suicidality were rare and were similar between the montelukast and placebo or active-control groups [99]. At present, there is insufficient data to prove that there is a link between montelukast and suicidality [100]. Results from three randomized, double-masked, controlled trials conducted by the American Lung Association Asthma Clinical Research Centers, that included a total of 1469 patients of whom 569 patients were assigned to montelukast, did not show evidence of a negative effect of montelukast on emotional well being as a marker for depression [101]. However, due to the relevance of this safety issue, a close monitoring of suicidality in patients treated with LTRAs is warranted. There are limited prospective, comparative studies examining the safety of CysLT_1_ receptor antagonists in pregnancy [102]. Montelukast does not appear to increase the baseline rate of major malformations [102,103]. The lower birth weight observed in infants born to women treated with montelukast could be attributed to severity/control of the maternal asthma [102,103]. 

Oral administration of CysLT_1_ receptor antagonists provides a single therapeutic approach to allergic rhinitis and asthma. In asthmatic patients with allergic rhinitis, a combined treatment approach that includes montelukast and budesonide is more effective in reducing airflow obstruction compared with doubling the dose of budesonide, indicating that this strategy increases therapeutic efficacy potentially reducing the number of side effects of inhaled glucocorticoids [104].

## 7. Conclusions

Most of our knowledge of the pathophysiological role of LTs in asthma is currently limited to CysLT_1_ receptor-mediated effects, whereas the role of the CysLT_2_ receptor is largely unknown. The identification of responders to CysLT_1_ receptor antagonists might be relevant for a more rational therapy of patients with asthma. In responders, CysLT_1_ receptor antagonists provide a therapeutic alternative to inhaled glucocorticoids in patients with persistent mild asthma. However, CysLT_1_ receptor antagonists are generally less effective than inhaled glucocorticoids. In patients with more severe asthma who respond to CysLT_1_ receptor antagonists, the addition of these drugs to inhaled glucocorticoids improves asthma control and enables the dose of inhaled glucocorticoids to be reduced while maintaining similar efficacy. The potential effect of CysLT_1_ receptor antagonists in preventing and reversing structural changes that characterize airway remodeling, as well as the role of LTB_4_ in asthma, requires further study.
